# White matter microstructural biomarkers predict cochlear implant outcomes in adults with postlingual deafness

**DOI:** 10.1093/braincomms/fcag373

**Published:** 2026-10-08

**Authors:** Anaïs M Grégoire, Nicolas Delinte, Laurence Dricot, Naïma Deggouj, Ron Kupers

**Affiliations:** Institute of Neurosciences (IoNS), Catholic University of Louvain (UCLouvain), 1200 Brussels, Belgium; Department of ENT, Academic Hospital of Saint-Luc, 1200 Brussels, Belgium; Institute of Neurosciences (IoNS), Catholic University of Louvain (UCLouvain), 1200 Brussels, Belgium; Institute of Neurosciences (IoNS), Catholic University of Louvain (UCLouvain), 1200 Brussels, Belgium; Department of ENT, Academic Hospital of Saint-Luc, 1200 Brussels, Belgium; Institute of Neurosciences (IoNS), Catholic University of Louvain (UCLouvain), 1200 Brussels, Belgium; École d’optométrie, Université de Montréal, H3T 1P1 Montréal, QC, Canada

**Keywords:** cochlear implant, brain plasticity, DWI, deafness, fractional anisotropy

## Abstract

Although cochlear implantation (CI) has been used for decades to restore hearing in sensorineural deafness, accurately predicting individual outcomes remains challenging—particularly in adults with postlingual deafness, a population that has been relatively understudied. In our previous work, we showed that speech comprehension after 6 months of CI use is better predicted when preoperative grey matter volume and cortical thickness in regions supporting language and cognition are considered alongside preoperative residual hearing and audiovisual abilities. In the present study, we investigated whether preoperative diffusion-weighted imaging (DWI) metrics in subcortical white matter (WM) could account for individual variability in cochlear implant adaptation and enhance predictive accuracy. Thereto, we collected preoperative DWI and speech performance under auditory-only and audiovisual conditions in 65 adults with postlingual bilateral deafness. Improvement in speech comprehension with CI was evaluated at 6 and 12 months post-implantation. A whole-brain analysis revealed significant positive correlations between preoperative fractional anisotropy (FA) within a cluster in the left arcuate fasciculus and speech perception at 6 months in both auditory-only and audiovisual conditions. Incorporating FA from the arcuate fasciculus cluster into the multiple linear regression model developed in our previous study significantly improved prediction accuracy for CI outcomes at 6 months. We also identified FA clusters in the right inferior fronto-occipital fasciculus and the corpus callosum that showed positive relationships with audiovisual comprehension and mid-term cochlear implant performance. In contrast, mean FA values averaged across entire tracts did not show similarly strong associations with speech perception, as shown after tract reconstruction. Notably, sex modulated the relationship between age and FA values across multiple white matter clusters. These findings underscore the role of localized WM microstructural properties in shaping speech adaptation after CI. Identifying WM-based predictors of CI performance may aid clinicians in forecasting individual outcomes and tailoring rehabilitation strategies, particularly for patients at risk of delayed or limited benefit. Further research is needed to clarify the origins of regional FA variability.

## Teaser

Grégoire *et al*. show that preoperative diffusion-weighted MRI measures, particularly fractional anisotropy, improve prediction of speech comprehension after cochlear implantation in postlingually deaf adults. Associations vary by brain region, postoperative time point and speech condition (auditory-only versus audiovisual), highlighting the role of white matter microstructure in individual outcome variability.

## Graphical Abstract

**Graphical Abstract:**
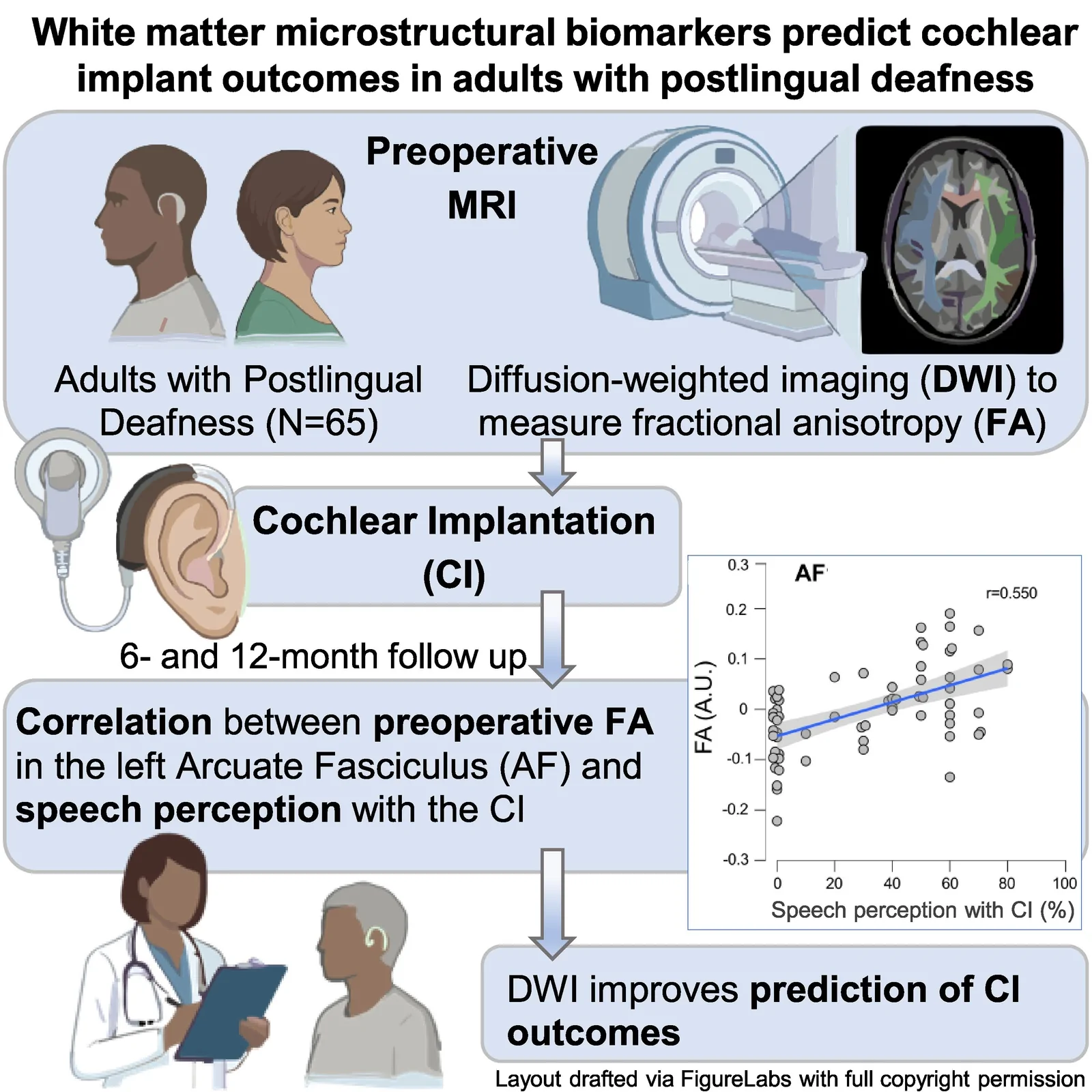

## Introduction

Acquired or postlingual deafness represents a significant and growing healthcare challenge in our ageing populations.^1^ Cochlear implantation has become the treatment of choice for individuals with severe to profound sensorineural hearing loss (HL), with more than 1 million cochlear implants (CIs) placed worldwide as of July 2022.^2^ While long-term outcomes are generally favourable, there remains considerable variability in individual results.^3^ Numerous factors are known to influence outcomes, including the duration of auditory deprivation, the aetiology of deafness, variability in genetic aetiology, the depth of electrode insertion and individual differences in preoperative linguistic and cognitive abilities.^3-7^

Brain plasticity also plays a role in the success of CI rehabilitation.^8-13^ Neuroplastic changes occur both in congenital and acquired auditory deprivation, producing persistent effects on brain structure and function.^14,15^ Such cerebral modifications may be due to the sensory deprivation itself, or to compensatory coping mechanisms, such as the acquisition of sign language in individuals with congenital deafness, or the development of lip-reading skills in those with acquired HL.^16^ MRI studies *in vivo* have revealed structural brain changes not only in congenitally deaf individuals, which is the most studied deaf population, but also in patients with acquired deafness or milder forms of HL, especially in age-related HL.^14,16-18^ The observed changes involve widespread regions of both grey matter (GM) and white matter (WM).^11,14,16^ Extending beyond regional volumetric changes in structural WM, diffusion-weighted imaging (DWI) methods can reveal more subtle microstructural changes in WM tracts. The metrics obtained through DWI depict aspects of water diffusion in organized fibre bundles, more specifically fractional anisotropy (FA), axial diffusivity (AD), radial diffusivity (RD) and mean diffusivity (MD). Among these, FA is the most extensively studied parameter,^16^ reflecting the degree of directional water diffusion within a voxel, which serves as an indirect marker of the polarized WM microstructure. Higher FA values typically indicate aligned axonal organization, as seen in healthy WM tracts, where water diffusion is constrained along a single axis by the surrounding myelin sheaths of parallel axons.^19^

Several studies have shown that deafness is associated with significant changes in WM.^14,17,20,21^ Individuals with congenital or acquired forms of deafness have decreased FA and increased RD along the entire auditory connectome, including the inferior colliculus, the lateral lemniscus and post-thalamic radiations,^17,20-25^ extending towards the auditory cortex in the superior temporal gyrus,^14,20-23,26^ and further into the frontal lobes,^16,26,27^ especially around the left inferior frontal gyrus.^20,21^ Modifications in WM tracts involved in language processing have been reported even in cases of mild to moderate acquired HL.^20,23,28-30^ Such findings include decreased FA and increased MD or RD within the superior longitudinal fasciculi (SLFs),^20,23,28,29,31^ the uncinate fasciculi,^25,28,31^ the inferior longitudinal fasciculi and the inferior fronto-occipital fasciculi (IFOFs), especially in individuals with moderate and age-related HL.^23,25^ Furthermore, alterations of the corpus callosum (CC) have been reported in both congenital^22,26,32,33^ and acquired deafness, even in cases of moderate HL,^20,22,25,29,30^ with main changes in the body and splenium, including the forceps major.

Brain structure and function can vary substantially among deaf individuals, which is likely to influence CI outcomes. For example, congenitally deaf children, and likewise adults with postlingual deafness, with greater preoperative activity at rest in the left superior fronto-parietal region tend to achieve better CI performance.^8,11^ While studies in congenitally deaf individuals have established that structural characteristics can impact CI outcomes^21,34^ as well, there are relatively few corresponding investigations in adults with postlingual deafness. Of note, two recent studies, including one from our group, reported a positive correlation between speech perception outcomes following CI and increased GM volume and cortical thickness in the left superior temporal cortex and left inferior frontal gyrus.^13,35^ Much less is known about the contribution of the WM structure to CI outcomes in adults with postlingual deafness, which is a matter amenable to study by DWI methods.^18^

The objective of the present study was therefore to explore the relationship between WM microstructure and speech perception abilities after CI in postlingually deaf adults. To address this question, we collected preoperative structural and DWI data along with clinical data in 65 CI patients. This research involves largely the same cohort as a previous study that focused on predicting audiovisual speech perception scores (AVs) at 6 months post-activation from GM metrics and clinical data.^35^ We now extend that analysis to include both short- and mid-term outcomes (at 6 and 12 months), examining performance in both auditory-only and audiovisual conditions, with the introduction of DWI-derived measures as additional outcome predictors. Since most CI patients experience their greatest improvements in the first 3–6 months following implantation, the 6-month assessment largely reflects the rate of early auditory adaptation.^36^ In contrast, speech perception by 12 months post-activation provides a reliable estimate of mid-term CI performance, capturing also outcomes from individuals with slower CI adaptation and those who had made only limited use of their device in the first post-surgical months. We conducted a whole-brain analysis to ascertain the relationship between preoperative FA metrics and CI outcomes. Furthermore, we investigated the roles of confounding factors, such as residual hearing, duration of HL, handedness and sex.

## Materials and methods

### Patients

The study population consists of 71 adults with postlingual bilateral deafness, who had been recruited at our tertiary academic hospital between 2018 and 2022 (Fig. 1). All patients met the national criteria for cochlear implantation, as defined by the National Institute for Health and Disability Insurance, which requires hearing thresholds of ≥70 dB HL at three of the four following frequencies: 0.5, 1, 2 and 4 kHz (https://webappsa.riziv-inami.fgov.be/simpl/). Patients who were not native or fluent French speaking were excluded from the study, as limited proficiency in French could affect speech comprehension outcomes. Individuals with psychiatric or neurological co-morbidities, as well as those with other disabling sensory impairments, were also excluded from the study. After screening, there remained 65 CI patients with preoperative MRI and recording of CI outcomes at 6 months post-activation, and 63 patients at 12 months. Written informed consent was obtained from each patient, and the study was approved by the local ethics committee of the hospital and faculty (2017/02AOU/384).

**Figure 1:**
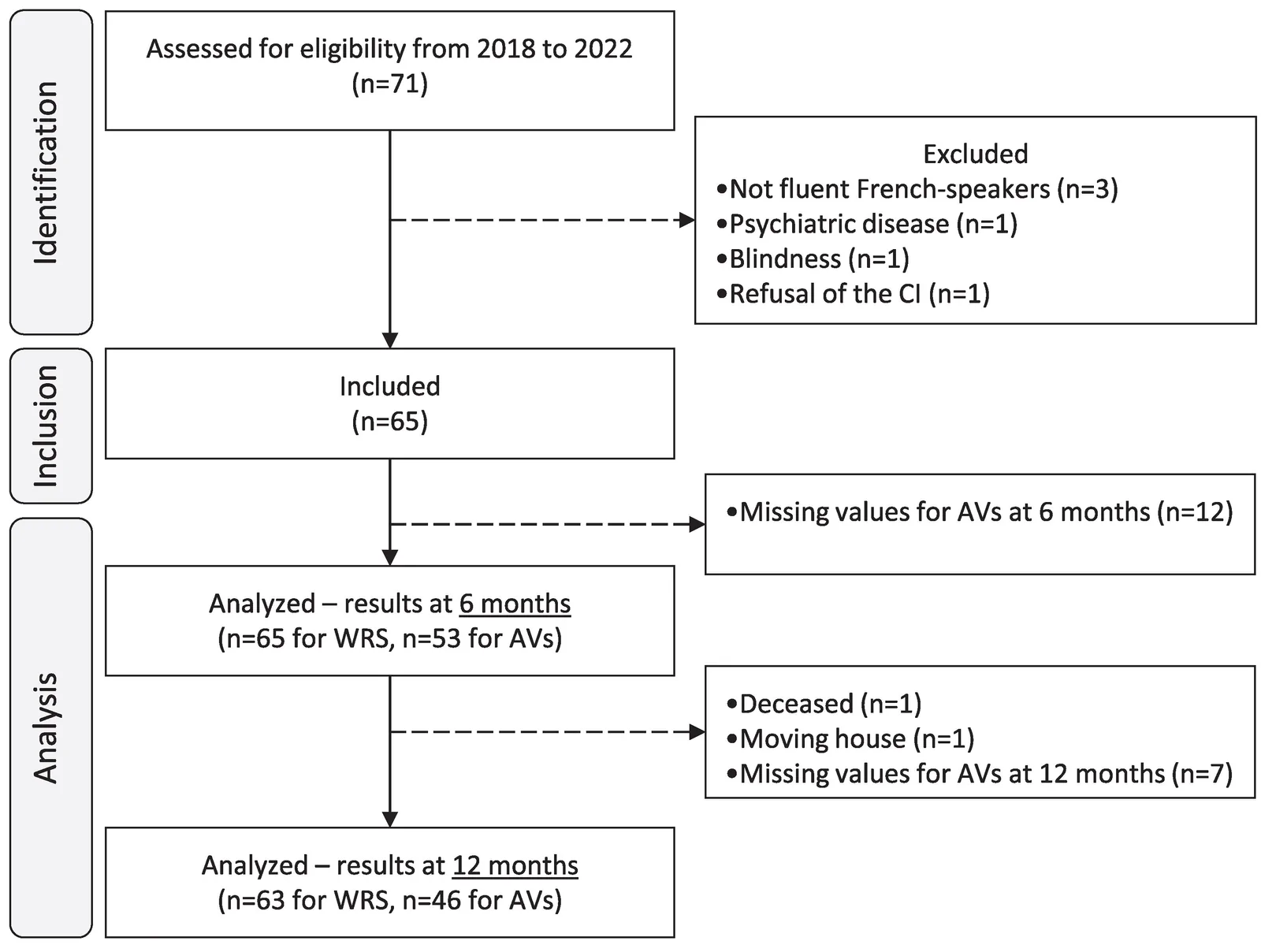
**Flowchart of the study design.** Abbreviations: C, cochlear implant; WRS, word recognition score; AVs, audiovisual speech perception score.

### Preoperative behavioural measures

In addition to age, sex and handedness, we recorded a range of preoperative demographic and clinical variables, such as hearing and speech perception scores and the patients’ history of hearing-aid (HA) use. We used the age of HA onset as an indicator of the onset of disabling HL. Depression and anxiety symptoms were measured using the Hospital Anxiety and Depression Scale (HADS) questionnaire.^37^ Unaided hearing was assessed using the pure-tone average [pure tone audiometry (PTA), dB HL], calculated from thresholds at 0.5, 1, 2 and 4 kHz for each ear. Speech perception was evaluated in an open field using the Lafon monosyllabic word lists.^38^ Stimuli were delivered through a loudspeaker positioned directly in front of the participant or through two loudspeakers placed laterally. Word recognition was tested at presentation levels of 40, 55 and 70 dB sound pressure level (SPL), and the mean word recognition score (WRS) across the three intensities was used for analysis. In the aided condition, participants wore their HAs according to their usual daily settings. AVs was assessed using a locally developed test.^39^ During the preoperative evaluation, this assessment was conducted with participants in their optimal aided condition while wearing their HAs. The AVs consists of audiovisual recordings of a female speaker presenting monosyllabic French words selected from two validated and balanced lists of 10 items each. Participants were instructed to repeat each word they recognized. Testing was performed in the audiovisual condition, allowing the use of both auditory input and speechreading cues. The stimuli were presented at 70 dB SPL in a clear speaking voice, and performance was expressed as the percentage of correctly identified words.

### Cochlear implant outcome

The outcome of CI therapy was evaluated at 6 and 12 months post-activation (i.e. 7 and 13 months after surgery). Postoperative WRS and AVs were measured with the CI in an open-field setting in most cases, without the use of a contralateral HA when applicable. In cases with residual hearing in the contralateral ear, the OtoCube system (Otocube Limited, Geertruidenberg, the Netherlands) was employed. This system consists of a soundproof box equipped with a loudspeaker, serving as a substitute for a conventional audiological booth.^40^ The CI processor is placed inside the box and connected via a long cable to the coil positioned on the patient’s head. The inclusion of both auditory-only and AVs scores reflects complementary assessment goals. Auditory-only measures isolate peripheral hearing function and the fidelity of auditory signal processing provided by the CI, offering a direct index of acoustic input quality.^41^ In contrast, AVs relies on broader cognitive processes, integrating visual articulatory cues to complement degraded auditory input.^42,43^ As speech in everyday life is typically audiovisual and conversational, these conditions are more ecologically valid and better reflect the real-world communication demands faced by CI users.^41,42,44-46^ Furthermore, AVs appears to better reflect real-life communication abilities and correlates more strongly with CI-related quality of life than isolated WRSs.^47^

Information on the type of electrodes used (straight versus perimodiolar) and the side of implantation (right or left) was also recorded.

### Preoperative MRI

MRI data were acquired on a 3 Tesla Skyra MRI scanner (Siemens Healthcare, Erlangen, Germany) equipped with a 20-channel phased-array head-neck coil. The imaging protocol included the following sequences: a 3D T1-weighted structural scan (acquisition parameters as detailed in Grégoire *et al.*^35^), DWI and resting-state functional MRI. However, results from the resting-state data are not reported herein. Diffusion imaging parameters were as follows: repetition time = 9000 ms, echo time = 74 ms, 64 diffusion-weighted directions with a *b*-value of 800 s/mm^2^ and one non-diffusion-weighted (*b* = 0 s/mm^2^) volume. The field of view was 256 × 256 mm^2^, with a matrix size of 128 × 128, resulting in an isotropic voxel size of 2 × 2 × 2 mm^3^ (slice thickness = 2 mm, 56 contiguous slices, no interslice gap).

Preprocessing of the diffusion data was performed using the in-house developed pipeline ‘ElikoPy’.^48^ In the absence of a reversed phase-encoding b0 image, a distortion-free unweighted diffusion image was synthesized from the T1-weighted anatomical scan using Synb0-DisCo.^49^ The pipeline included brain extraction,^50^ thermal noise removal,^51^ susceptibility distortion correction^52^ and correction for eddy currents and head motion. A diffusion tensor model^53^ was then fitted using DIPY,^54^ whereupon FA maps were generated and normalized to Montreal Neurological Institute (MNI) space.

A whole-brain analysis was then conducted using BrainVoyager (Brain Innovation, version 23.2). We employed an analysis of covariance (ANCOVA) to identify potential correlations between FA maps and speech perception with CIs at 6 and 12 months post-activation, during the auditory-only (WRS) and the audiovisual (AVs) conditions. Speech perception scores were adjusted for age using a regression model to account for the confounding effect of age. The ANCOVA was conducted with an uncorrected *P*-value of 0.001. Monte Carlo simulation was then employed to determine a cluster-level statistical threshold following 3000 permutations. eBrains was used to identify WM fibre bundles (EBRAINS Knowledge Graph, ‘https://kg.ebrains.eu’).

In addition to the whole-brain analysis, we conducted tractography of specific tracts of interest to investigate the link between streamline count or mean FA value across the whole tract and CI outcomes. Tract selection was guided by the results of the whole-brain analysis, focusing on the bilateral arcuate fasciculus (AF), IFOF and the CC. The regions of interest (ROIs) used as seeds and targets were extracted from the Automated Anatomical Labelling atlas, defined in MNI space,^55^ MNI152, and dilated to include the WM boundary. The inclusion ROIs in the WM were manually drawn in MNI space using MRIcroGL based on anatomical markers.^56^ All ROIs were then registered to each patient’s native space through a two-step transformation process. More precisely, the ROIs were first aligned from MNI to the patient’s T1-weighted anatomical image, and then from the T1 to the diffusion space. The CC was subdivided into five segments^57^: genu, anterior and posterior midbody, isthmus and splenium. Tractography was then performed on the preprocessed diffusion data using an ROI-based approach. Streamlines were generated using the tckgen command from the MRtrix3 toolbox, employing the probabilistic algorithm second-order Integration over Fiber Orientation Distributions for local fibre orientation modelling.^58^ The following main tracking parameters were used for each tract: cut-off = 0.1, maximum angle = 15°, maximum length = 200 mm and maximum number of streamlines = 2000. Once the tracts were generated, a filtering process was applied to eliminate aberrant or isolated streamlines using the Python library ‘Unravel’.^59-61^ The mean FA was then extracted for each individual tract, assigning a greater weight to voxels with higher streamline density to minimize the influence of stray streamlines.^61^

### Statistics

Comparisons of pre- and postoperative speech comprehension scores at 6 and 12 months after CI activation were performed using ANOVA, or a Friedman test in case of deviation from normality (assessed by the Shapiro–Wilk test). For each participant, FA values were extracted from the significant clusters identified in the whole-brain analysis as well as from the whole tracts studied by tractography. Partial correlations, controlling for age, were then performed between the FA values and clinical measures to investigate potential associations. Normality was assessed with the Shapiro–Wilk test. Pearson correlations were used for normally distributed variables, whereas Spearman correlations were applied when normality assumptions were not met. To correct for multiple comparisons and control the false discovery rate, we applied the Benjamini–Hochberg procedure, with a significance level set at *P* < 0.05.

We then conducted multiple linear regression analysis to examine the effects of handedness, age and sex on the non–age-corrected FA values. Age was included as a continuous predictor, and sex and handedness were categorical variables. An interaction term between age and sex (age × sex) was added to the model to test whether the association between age and FA varied across sex. All variables were entered simultaneously into the regression model.

Finally, we performed multiple linear regression analyses to evaluate the accuracy of CI outcome prediction based on preoperative variables. We extended the predictive model developed in our earlier study,^35^ which used preoperative measures to estimate AVs at 6 months, by applying it to outcomes at 12 months and incorporating, as an additional covariate, the FA of the WM regions exhibiting the strongest association with CI results. Model performance was assessed using adjusted *R*^2^.

All statistical analyses were performed using JASP version 0.19.3,^62^ except for the linear regression models, which were conducted using SPSS version 25 to ensure consistency with our previous study.^35^

## Results

### Patient characteristics

Table 1 shows the patients’ demographic characteristics. Fifty-five per cent of the patients were females, and 85% were right-handed (10% left-handed and 5% ambidextrous). Nearly all patients (97%) wore HAs before CI, with 71% wearing HAs in both ears. Twenty-one per cent of the patients had stopped wearing HAs in the ear that was subsequently implanted, due to an absence of further therapeutic gain. Seven patients had a history of progressive childhood-onset HL and wore hearing aids for mild to moderate uni- or bilateral impairment before age 7, yet all demonstrated correct oral language development. Some patients presented a ‘ski-slope’ HL, with preserved residual hearing at low frequencies (as good as 25 dB HL at 0.5 kHz) but profound HL at high frequencies.

**Table 1:** Demographic characteristics and behavioural results

		Mean ± SD	Median	Range	Frequency
Age (years)		57.5 ± 18.4	62	18–88	
Sex					55% female
Handedness					55 (85%) right-handed, 7 (10%) left-handed, 3 (5%) ambidextrous
DHA (years)		17.6 ± 12.5	15	1–63	
Onset HL (years)		41 ± 21	42	5–77	
HADS—anxiety a		8 ± 4	7	1–16	54% normal, 27% borderline, 19% abnormal
HADS—depression a		5 ± 3	5	0–15	77% normal, 15% borderline, 8% abnormal
PTA (dB HL)	CI side	99 ± 14	99	70–120	
	Non-CI side	91 ± 16	88	65–120	
HT 0.5 kHz (dB HL)	CI side	89 ± 26	95	25–120	
	Non-CI side	77 ± 25	75	20–120	
WRS (%)	W/o HA	3 ± 10		0–60	
	W/HA	17 ± 22		0–90	
AVs (%)		48 ± 23		0–100	

Abbreviations: AVs, audiovisual speech perception score; DHA, duration of HA use; onset HL, age at onset of HL; PTA, pure tone audiometry; HT 0.5 kHz, hearing thresholds at 0.5 kHz; WRS, average word recognition score; HL, hearing loss.

^a^HADS anxiety and depression normative scores: 0–7, normal; 8–10, borderline; and 11–21, abnormal.

The right ear was implanted in 60% of the cases, with no statistically significant difference from an equal distribution (binomial test). Forty-eight per cent of the CIs were from Cochlear®, 29% from MED-EL® and 23% from Advanced Bionics®. Perimodiolar and straight electrodes were placed in 51% and 49% of the surgeries, respectively.

### Cochlear implant outcome

As expected, speech perception improved significantly following CI implantation compared to preoperative speech scores obtained with HAs only (Friedman test, *P* < 0.001 for WRS and *P* = 0.002 for AVs) (Fig. 2). WRS improved significantly across all conditions (Conover’s *post hoc* comparisons, *P* < 0.001 after Holm correction for multiple comparisons). *Post hoc* analysis also showed a significant improvement in AVs between the preoperative assessment with HAs and the postoperative assessments with the CI only at 12 months after CI activation (Conover test, *P* = 0.001 after Holm correction for multiple comparisons).

**Figure 2:**
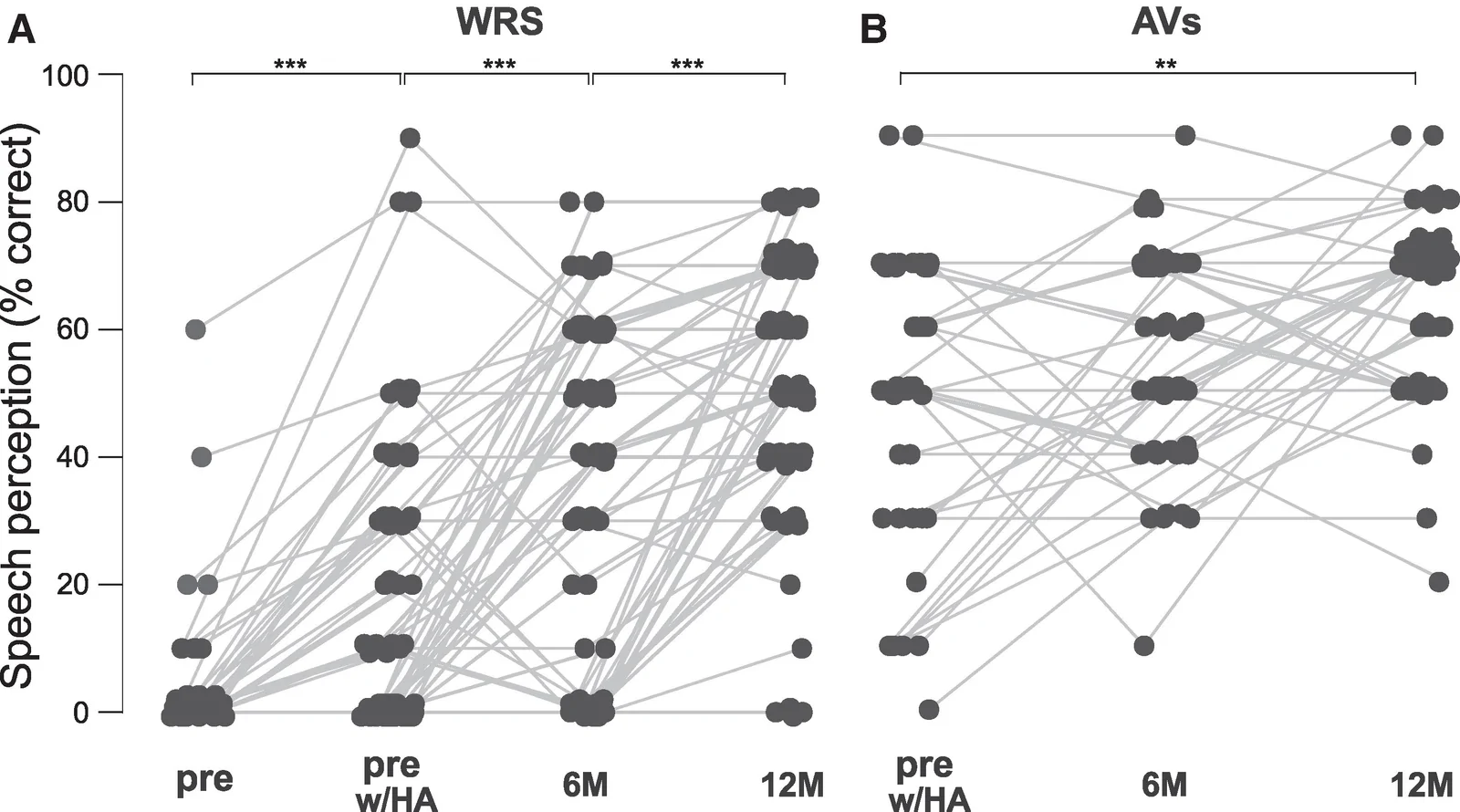
**Improvement of speech perception following CI implantation.** Speech perception scores in the auditory-alone condition (WRS, **A**) and audiovisual condition (AVs, **B**) are shown for each subject as individual data points, assessed preoperatively without HAs (pre), preoperatively with HAs (pre w/HA) and at 6 and 12 months after CI activation using the CI alone (6 and 12 M, respectively). Each line connects repeated measurements from the same participant. Speech perception improved significantly following CI implantation compared with preoperative scores obtained without and with HAs (Friedman test, *P* < 0.001 for WRS and *P* = 0.002 for AVs). Significant differences identified by Conover’s *post hoc* comparisons are indicated by asterisks, after Holm correction for multiple comparisons (**P < 0.010, ***P < 0.001). Data are based on *N* = 65 for the measures of WRS pre, WRS w/HA, WRS at 6 months and AVs, *N* = 63 for WRS at 12 months, *N* = 53 for AVs at 6 months and *N* = 46 for AVs at 12 months. Abbreviations: CI, cochlear implant; WRS, word recognition score; AVs, audiovisual speech perception score; pre, preoperatively; pre w/HA, preoperatively with hearing aids.

At 6 months post-CI activation, WRS and AVs were strongly correlated (*r* = 0.628, *P* < 0.001), whereas this association was not observed at 12 months (*r* = 0.224, *P* = 0.135). Furthermore, speech scores at 6 and 12 months were positively correlated, with a strong correlation for WRS (*r* = 0.632, *P* < 0.001) and a weaker, borderline non-significant correlation for AVs (*r* = 0.291, *P* = 0.052).

Nevertheless, CI outcomes demonstrate considerable variability between individuals, with WRS ranging from 0 to 80% with the CI. Correlation analysis between CI outcomes and various preoperative measures revealed that age negatively impacted auditory-alone speech perception at 12 months (*r* = −0.514, *P* < 0.001), with a lesser, though still strong, impact of age of onset of HL (*r* = −0.369, *P* = 0.003) (Fig. 3). Duration of HA use, taken as an indirect measure for the duration of HL, was not correlated with age or with CI outcomes. There was a significant effect of age on AVs measured at 12 months (*r* = −0.370, *P* = 0.011).

**Figure 3:**
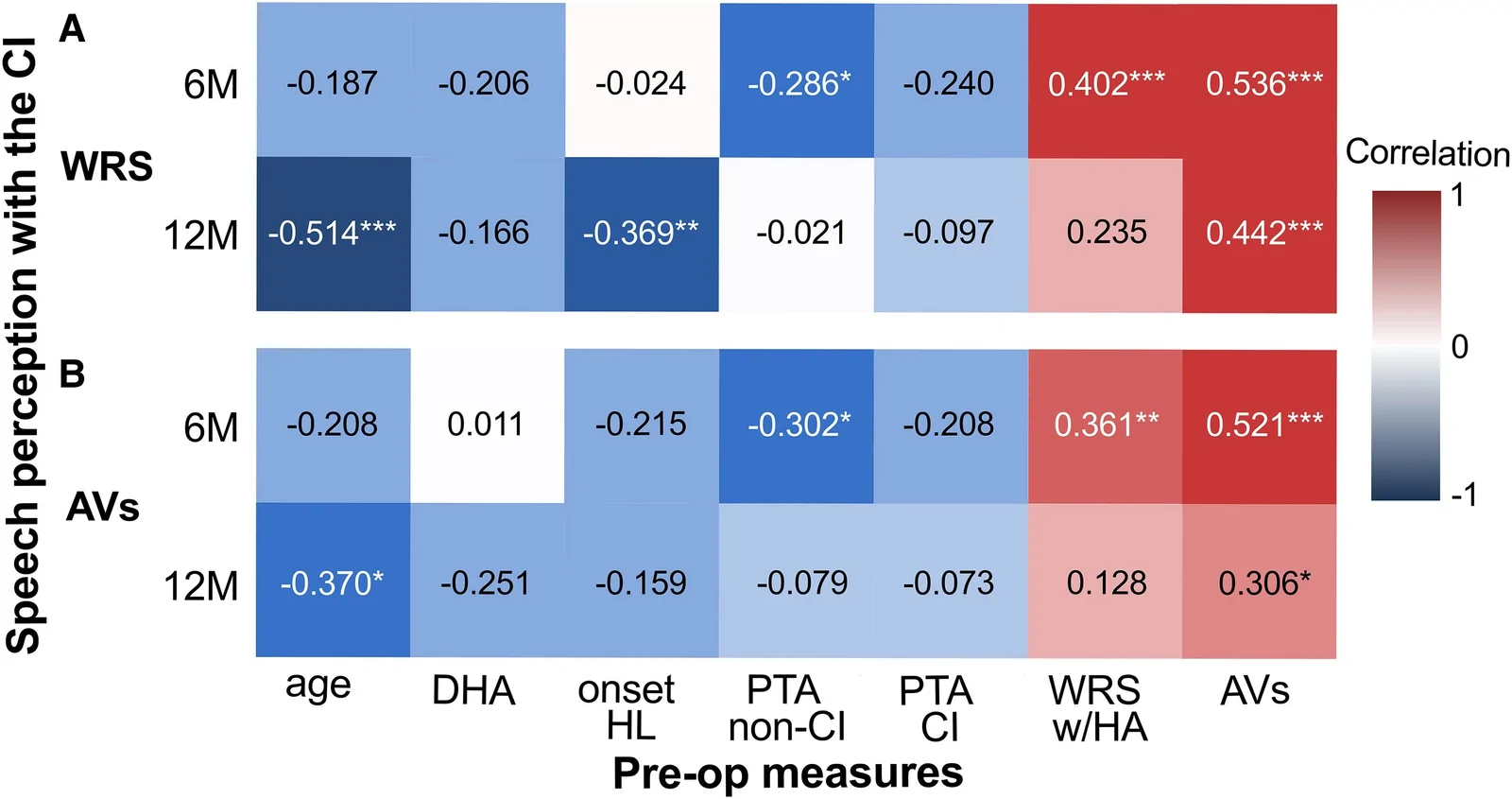
**Correlation matrix of speech perception with the CI and preoperative demographic and behavioural data.** Speech perception with the CI was recorded in the auditory-alone (WRS, **A**) and audiovisual conditions (AVs, **B**), at 6 (6 M) and 12 months (12 M) post-activation. The coefficients of correlation are reported, with asterisks highlighting significant results (Spearman’s correlation, *P < 0.050, **P < 0.010, ***P < 0.001). The color coding emphasizes the correlation coefficients: red indicates positive relationships, while blue indicates negative relationships, with higher color saturation reflecting a stronger relationship. Data are based on *N* = 65 for correlations with WRS at 6 months, *N* = 63 for WRS at 12 months, *N* = 53 for AVs at 6 months and *N* = 46 for AVs at 12 months. Abbreviations: CI, cochlear implant; WRS, word recognition score; AVs, audiovisual speech perception score; pre, preoperatively; pre w/HA, preoperatively with hearing aids; DHA, duration of hearing-aid use; HL, hearing loss; PTA CI side/PTA non-CI side, pure tone audiometry on the side that was later implanted/was not implanted.

Furthermore, speech perception with the CI correlated with preoperative speech perception. Preoperative hearing thresholds in the non-implanted ear (PTA non-CI) were associated with speech outcomes at 6 months for WRS (*r* = −0.286, *P* = 0.021) and for AVs (*r* = −0.302, *P* = 0.026), indicating that lower hearing thresholds (i.e. better residual hearing) give rise to better CI outcomes. Moreover, preoperative WRS with HAs showed significant correlations with auditory (*r* = 0.402, *P* < 0.001) and audiovisual speech comprehension (*r* = 0.361, *P* = 0.007) at 6 months, while preoperative AVs was associated with CI outcomes at both 6 (*r* = 0.536 for WRS and *r* = 0.521 for AVs, *P* < 0.001) and 12 months (*r* = 0.442, *P* < 0.001 for WRS and *r* = 0.306, *P* = 0.041 for AVs). As expected, higher preoperative speech perception with HAs (both WRS w/HA and AVs) was associated with better preoperative hearing thresholds in the non-implanted ear (*r* = −0.384, *P* = 0.002 for WRS and *r* = −0.319, *P* = 0.011 for AVs). In other words, better residual hearing on the non-implanted side (lower PTA at non-CI side) is important for better preoperative speech perception with HAs (WRS w/HA and AVs), which favoured faster CI rehabilitation (WRS and AVs, especially at 6 months).

Patients with more depressive symptoms had worse preoperative speech perception (*r* = −0.288, *P* = 0.038 for WRS w/HA) and lower auditory speech perception scores at 6 months post-CI (*r* = −0.320, *P* = 0.021). On the contrary, anxiety scores were not significantly correlated with speech perception, neither preoperatively (*P* = 0.997 for WRS w/o HA, *P* = 0.151 for WRS w/HA and *P* = 0.130 for AVs) nor after CI implantation (*P* = 0.095 and *P* = 0.468 for WRS, and *P* = 0.670 and *P* = 0.736 for AVs, at 6 and 12 months post-CI activation). There was a weak, non-significant negative correlation between both anxiety and depression and both age (*r* = −0.226, *P* = 0.107 for anxiety and *r* = −0.213, *P* = 0.129 for depression) and duration of HL (*r* = −0.263, *P* = 0.060 for anxiety and *r* = −0.204, *P* = 0.147 for depression). There were no significant differences in CI outcomes based on the side of implantation (Mann–Whitney test), with *P*-values of 0.052 and 0.203 for WRS and 0.301 and 0.747 for AVs at 6 and 12 months post-CI activation, respectively.

### Whole-brain diffusion-weighted imaging

The whole-brain analysis revealed several clusters of significant correlations between preoperative FA values and CI outcomes (Table 2; Figs. 4 and 5). At 6 months post-CI, auditory speech perception (WRS) correlated with FA of a cluster of 80 voxels belonging to the left AF within the left parietal lobe (AF1, Spearman’s correlation, *r* = 0.550, *P* < 0.001 Monte Carlo-corrected). For AVs, we found one cluster that correlated with FA in the CC around the forceps major (CC1, 84 voxels, Spearman’s correlation, *r* = 0.523, *P* < 0.001 Monte Carlo-corrected), and another one within the right IFOF (rIFOF; 85 voxels, Spearman’s correlation, *r* = 0.572, *P* < 0.001 Monte Carlo-corrected). A smaller cluster in the left AF did not survive Monte Carlo correction, likely due to its limited spatial extent (AF2, 64 voxels, Spearman’s correlation, *r* = 0.598, *P* < 0.001). At 12 months post-CI, AVs scores correlated positively with FA in a large WM cluster of the CC, extending to the internal capsule, and the cortico-ponto-cerebellar and cortico-spinal bundles, extending into the right hemisphere (CC2, 151 voxels, Spearman’s correlation, *r* = 0.573, *P* < 0.001 Monte Carlo-corrected). In contrast, WRS correlated negatively with FA in a cluster within the parieto-frontal region, including the left cortico-spinal tract (CST) and the internal capsule (98 voxels, Spearman’s correlation, *r* = −0.444, *P* < 0.001 Monte Carlo-corrected).

**Figure 4:**
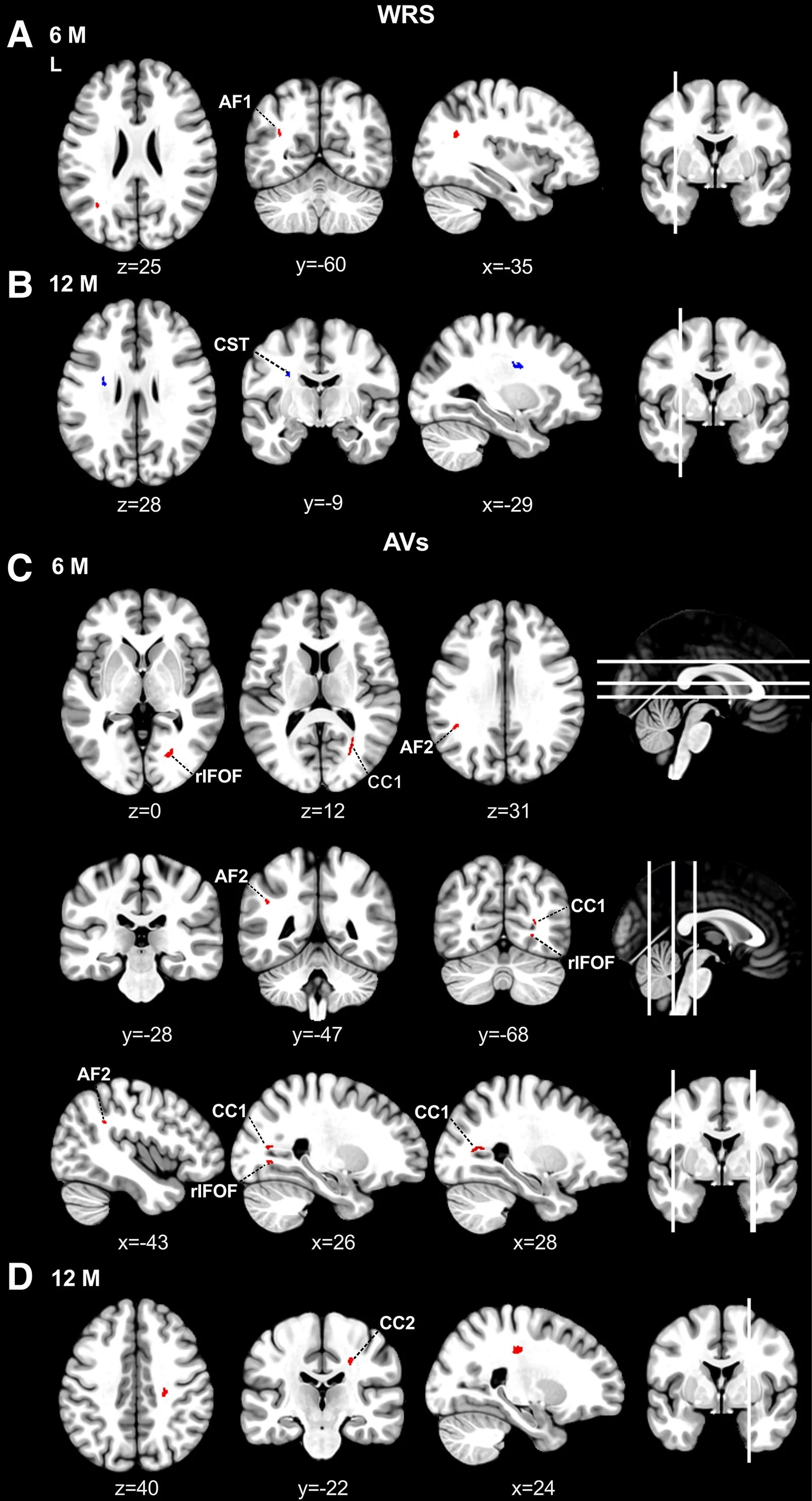
**Correlations between FA (whole brain) and CI speech perception in the auditory (WRS) and audiovisual (AVs) conditions.** The whole-brain analysis showed clusters with significant correlations between preoperative FA values and post-CI WRS (**A** and **B** for correlations with WRS at 6 and 12 months, respectively) and AVs (**C** and **D** for correlations with AVs at 6 and 12 months, respectively) (ANCOVA, *P* < 0.001 after Monte Carlo simulation). The clusters with positive correlations are represented in red, and the negative ones in blue. The *x*, *y* or *z* coordinates in MNI space are mentioned below each slice. The clusters (AF1, AF2, rIFOF, CST, CC1 and CC2) are named in accordance with the main text and Table 2. Data are based on *N* = 65 for correlations with WRS at 6 months, *N* = 63 for WRS at 12 months, *N* = 53 for AVs at 6 months and *N* = 46 for AVs at 12 months. Abbreviations: WRS, word recognition score; AVs, audiovisual score; AF, arcuate fasciculus; (r)IFOF, (right) inferior fronto-occipital fasciculus; CC, corpus callosum; CST, cortico-spinal tract.

**Figure 5:**
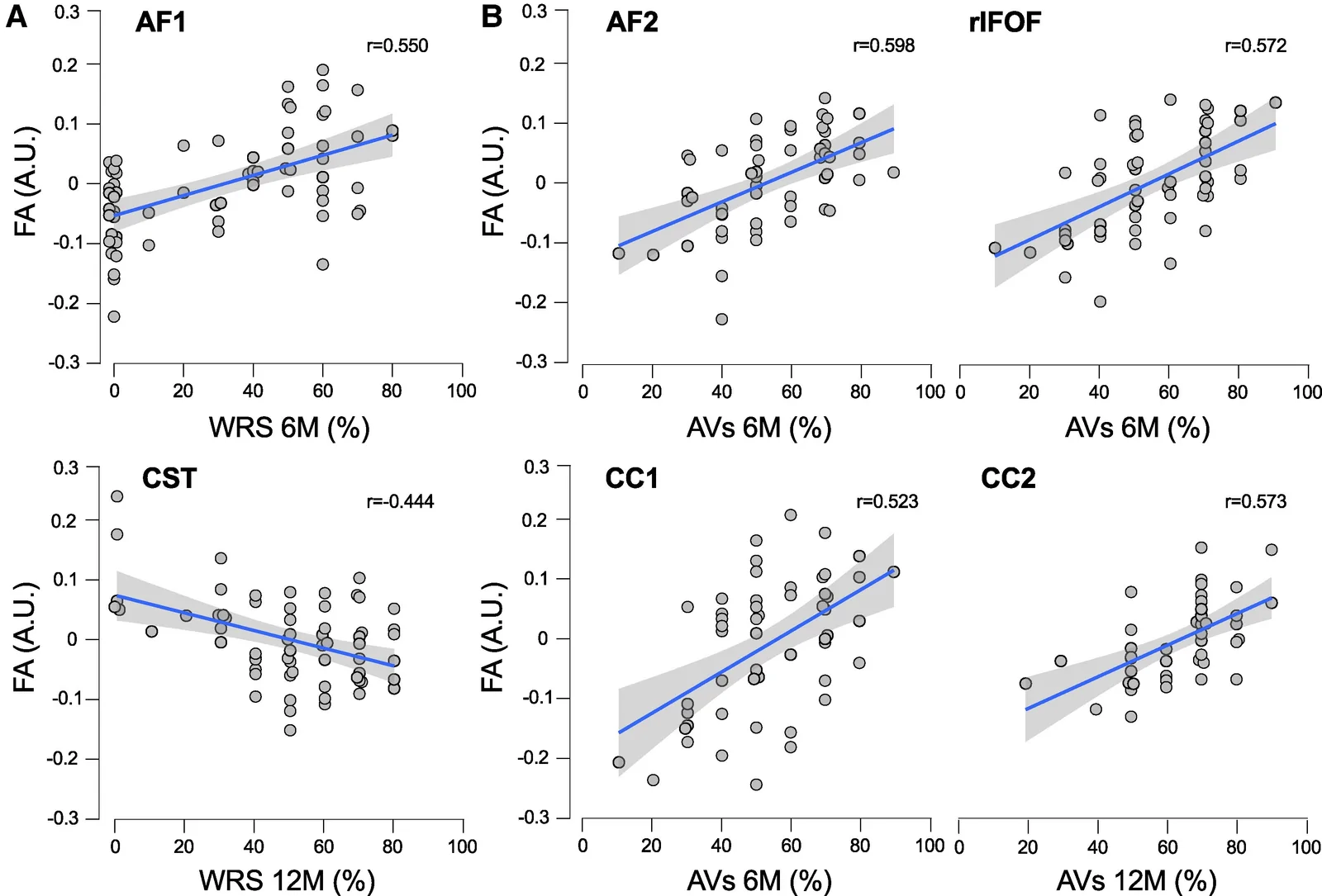
**Correlations between individual FA values for each significant cluster and speech perception with the CI.** The scatter plots illustrate the relationship between the average age-corrected FA value within each significant cluster identified in the whole-brain analysis (vertical axis) and speech perception scores (horizontal axis), in either the auditory (WRS, **A**) or audiovisual (AVs, **B**) conditions. Each dot represents an individual patient. FA values were adjusted for age using a regression model. Linear regression lines are displayed with 95% confidence intervals (blue-shaded areas). Correlation coefficients (*r*) between the mean FA value of each cluster and speech outcomes are shown (Spearman’s partial correlations controlling for age; *P* < 0.001). Cluster labels correspond to those described in the main text and Table 2. Data are based on *N* = 65 for correlations with WRS at 6 months, *N* = 63 for WRS at 12 months, *N* = 53 for AVs at 6 months and *N* = 46 for AVs at 12 months. Abbreviations: WRS, word recognition score; AVs, audiovisual speech perception score; FA, fractional anisotropy; A.U., artificial unit; AF, arcuate fasciculus; (r)IFOF, (right) inferior fronto-occipital fasciculus; CC, corpus callosum; CST, cortico-spinal tract.

**Table 2:** Significant correlations between FA and CI outcomes at 6 and 12 months

	MNI coordinates			Size	correlates		Mean FA within the cluster ± SD (range)
Cluster	*x*	*y*	*z*	(mm^2^)	with	*r*	
6 months post-CI							
AF1	−35	−60	24	80	WRS	0.550	0.42 ± 0.09 (0.17–0.60)
AF2	−44	−47	32	64*	AVs	0.598	0.44 ± 0.08 (0.23–0.60)
rIFOF	25	−69	−1	85	AVs	0.572	0.38 ± 0.08 (0.20–0.60)
CC1	27	−67	12	84	AVs	0.523	0.59 ± 0.11 (0.34–0.80)
12 months post-CI							
CC2	24	−22	37	151	AVs	0.573	0.39 ± 0.07 (0.21–0.63)
CST	−29	−7	30	98	WRS	−0.444	0.42 ± 0.09 (0.17–0.60)

Spearman’s correlations were significant at *P* < 0.001 for all clusters, and all survived Monte Carlo correction except cluster AF2, possibly due to its smaller size (highlighted by an asterisk). Data are based on *N* = 65 for correlations with WRS at 6 months, *N* = 63 for WRS at 12 months, *N* = 53 for AVs at 6 months and *N* = 46 for AVs at 12 months.

Abbreviations: WRS, word recognition score; AVs, audiovisual speech perception score; AF, arcuate fasciculus; (r)IFOF, (right) inferior fronto-occipital fasciculus; CC, corpus callosum; CST, cortico-spinal tract.

To verify that the relationships between FA values and CI outcomes were not largely attributable to preoperative factors, we extracted the FA values from each significant cluster and tested for possible spurious correlations with the preoperative measures. This led to only one significant correlation, between the FA of the cluster CC1 and the hearing threshold on the non-implanted side (*r* = −0.402, *P* < 0.050 false discovery rate (FDR)-corrected), which means that individuals with lower thresholds (i.e. better residual hearing) had higher FA scores.

Next, we conducted for each cluster a linear regression analysis to examine the effects of handedness, age and sex, and the interaction between age and sex, on the FA values uncorrected for age. Results showed that ambidextrous and left-handed patients demonstrated relatively lower FA in the left-sided CST cluster compared to right-handed patients (unstandardized coefficient *b* = 0.105, *P* = 0.025). There was no significant effect of age, sex or their interaction on the FA of clusters CC1, rIFOF and CST. On the other hand, the relationship between age and FA varied depending on sex for the clusters AF1 and CC2. Specifically, for AF1, age and the interaction between age and sex significantly impacted the FA (*b* = −0.003, *P* < 0.001 for age and *b* = 0.003, *P* = 0.013 for the interaction between age and sex). In males, age had a significant negative association with FA of cluster AF1 (*r* = −0.585, *P* < 0.001), whereas no such association was observed in females (*r* = −0.129, *P* = 0.453) (Supplementary Fig. 1). For the cluster CC2, sex and the interaction between age and sex significantly impacted the FA (*b* = 0.122, *P* = 0.027 for sex and *b* = −0.002, *P* = 0.039 for the interaction between sex and age). Females exhibited relatively higher FA in the CC2 cluster (FA = 0.437 ± 0.067 for females versus 0.422 ± 0.065 for males), with a significant age-related decline observed in this group but not in males (*r* = −0.422, *P* = 0.010 for females versus *r* = 0.069, *P* = 0.722 for males) (Supplementary Fig. 1).

### Tractography results

Following reconstruction of the AF, IFOF and CC tracts (Fig. 6), we investigated whether their microstructural properties correlated with speech comprehension outcomes after CI. Specifically, we examined correlations between streamline counts and the mean FA of each tract, on the one hand, and speech comprehension scores, on the other hand, in both auditory-only and audiovisual conditions after 6 and 12 months of CI use. At 6 months, AV scores showed a positive correlation with streamline count of the rIFOF (*r* = 0.377, *P* < 0.050 FDR-corrected). At 12 months, AV scores were positively correlated with streamline counts of the splenium of the CC (*r* = 0.530, *P* < 0.050 FDR-corrected). Furthermore, WRSs in the auditory-only condition at 12 months correlated with the streamline count of the left IFOF (*r* = 0.348, *P* < 0.050 FDR-corrected). Analyses based on the mean FA values of the whole tracts revealed fewer associations. The only significant correlation was observed between the FA of the genu of the CC and audiovisual scores at 6 months (*r* = 0.355, *P* < 0.050 FDR-corrected).

**Figure 6:**
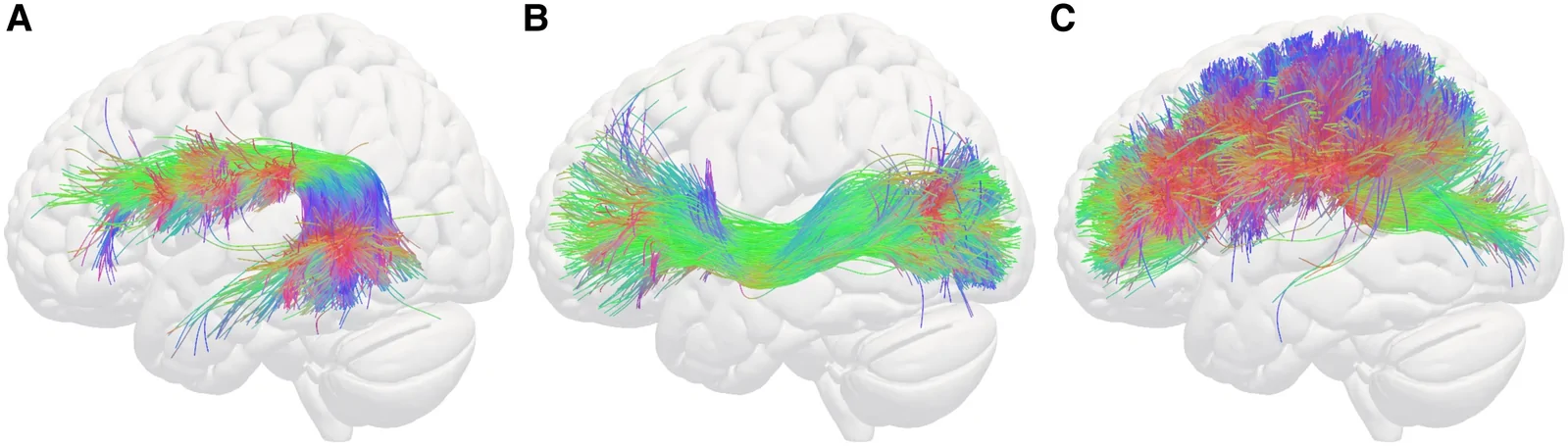
**Tract’s reconstruction.** The figure shows the left AF (**A**), the left IFOF (**B**) and the CC (**C**). Tractography is displayed using the standard orientation colour scheme (red, left–right; green, anterior–posterior; blue, superior–inferior). Data are based on *N* = 65.

### Linear regression models

We constructed a multivariate linear regression model to predict speech outcomes using the same preoperative variables previously identified by our group^35^: age at implantation, preoperative AVs, preoperative auditory-only speech perception with HAs (WRS w/HA), residual hearing threshold at 0.5 kHz in the non-implanted ear, depression score and cortical thickness of the left pars opercularis. Adding the mean FA value of the AF1 cluster, identified as the cluster most strongly correlated with speech perception in both auditory-only and audiovisual conditions at 6 months, significantly improved the model’s predictive performance, increasing the adjusted coefficient of determination (adjusted *R*^2^) from 0.493 to 0.564 (*P* < 0.001) (Supplementary Fig. 2 and Table 1). Although the initial model was designed to predict AVs rather than WRS, the same set of variables also significantly predicted WRS outcomes at 6 months, yielding an adjusted *R*^2^ of 0.479 (*P* < 0.001).

We next identified the optimal linear models for predicting outcomes at 12 months. Using the same variables as those included in the 6-month models resulted in limited predictive performance, with adjusted *R*^2^ values of 0.326 for WRS at 12 months (*P* = 0.002) and 0.183 for AVs at 12 months (*P* = 0.083), the latter not reaching statistical significance. We therefore developed optimized models to better predict 12-month outcomes. In these models, preoperative AVs, age and the FA value of AF1 remained significant predictors of both AVs and WRS at 12 months. Furthermore, the FA values of the CST and CC2 clusters emerged as additional significant predictors of WRS and AVs outcomes at 12 months, respectively, improving model performance (adjusted *R*^2^ = 0.495 for WRS and 0.502 for AVs; *P* < 0.001).

Finally, the FA values of all significant clusters identified in the whole-brain analysis (AF1, CC1, CC2, rIFOF and CST) were added to the set of predictor variables used in our previous study.^35^ The inclusion of the FA values improved the prediction performance of the models, estimating AVs at both 6 and 12 months (adjusted *R*^2^ = 0.689 and 0.550, respectively; *P* < 0.001). In contrast, predictive performance for WRS outcomes was lower, particularly at 12 months (adjusted *R*^2^ = 0.440, *P* < 0.001 for WRS at 6 months; adjusted *R*^2^ = 0.391, *P* = 0.002 for WRS at 12 months).

## Discussion

We conducted whole-brain diffusion imaging and tractography to test whether preoperative alterations in WM can help predict speech outcomes following CI implantation. The present study builds upon our previous work showing that the GM thickness and volume of brain areas involved in language processing (left pars opercularis and left superior and middle temporal gyri) and in cognition (superior frontal and superior parietal regions) relate to CI outcome at 6 months.^35^ In this follow-up study, we incorporated DWI measures in addition to the previously analysed GM features, and we also assessed speech outcomes at a longer postoperative period (12 months post-activation). At this time point, speech perception has stabilized for most CI recipients, allowing us to take into account individuals who needed more time to adapt to the implant, or who made insufficient use of the device during the initial months following implantation.

The whole-brain analysis revealed a positive correlation between FA of a cluster in the left AF and speech perception in both auditory-only and AV conditions. Additional FA clusters within the CC, the internal capsule and the rIFOF also showed significant positive correlations with AVs. Interestingly, we observed a negative correlation between FA in a cluster within the left CST and WRS at 12 months post-activation. Incorporating the FA values of the AF cluster into a linear regression model, together with clinical variables and preoperative cortical thickness of the left pars opercularis as established in our previous work,^35^ significantly improved the prediction of auditory and audiovisual speech outcomes at 6 months but not at 12 months. These findings suggest that determinants of mid-term CI outcomes may differ from those underlying the initial adaptation processes occurring during the first 6 months of device use. Among the original predictors, preoperative AVs remained significantly associated with outcomes at 12 months, while age was retained in the model to account for age-related brain changes. In addition, FA values of clusters located within the CC and CST further improved the predictive accuracy of the 12-month models. The observed differences in predictive factors for outcomes at 6 and 12 months may indicate ongoing neuroplastic reorganization induced by continued CI use. Certain factors, such as lower depression scores and greater cortical thickness of the left pars opercularis, may facilitate more rapid early adaptation to the CI. However, their influence appears to diminish over time, possibly because prolonged CI use promotes progressive neuroplastic reorganization that attenuates initial interindividual behavioural and anatomical differences.

When examining preoperative mean FA across the entire tracts (AF, IFOF and the five parts of CC) using tractography, the correlations with CI outcomes were smaller. This supports the hypothesis that CI performance is primarily associated with localized FA modifications, rather than with widespread, tract-wide WM changes. Similar patterns with focal rather than diffuse WM abnormalities have also been reported in conditions such as schizophrenia and Alzheimer’s disease.^63,64^ Conversely, increased streamline counts in certain reconstructed tracts were linked to better CI speech comprehension. These associations are examined more specifically in the sections that follow.

### The left arcuate fasciculus is associated with cochlear implant speech outcomes

Our findings suggest that the microstructural integrity within the left AF, which is part of the dorsal language stream, is critical for successful speech perception with a CI. The whole-brain analysis identified two clusters within the AF (AF1 and AF2), where FA correlated positively with WRS and AVs at 6 months. Additionally, the FA values of the AF1 cluster also correlated positively with WRS at 12 months. However, neither the mean FA of the entire AF nor its total streamline count correlated with CI outcomes. This suggests that localized microstructural alterations within specific segments of the AF, rather than global microstructural degradation or macrostructural changes, may be more relevant for explaining variability in speech outcomes.

The AF and adjacent SLF connect the posterior temporal and inferior parietal regions to the frontal inferior and premotor areas around the Sylvian fissure.^65,66^ These regions are collectively involved in phonological and syntactic processing, as well as auditory-motor integration.^66-72^ The activation of the dorsal phonological route, instead of the ventral one, and the higher activity within the left inferior (pre-)frontal cortex in response to speech have been shown to correlate with speech perception with a CI.^73-76^ This is corroborated by our current observations that higher FA within the AF predicted better hearing rehabilitation with the CI.

HL, whether congenital or acquired, has been shown to affect the microstructural features of the AF.^20,31^ Even mild or moderate loss in age-related^28^ or noise-induced HL^29^ can lead to decreased FA (and increased MD or RD), indicating altered microstructural organization. Certain protective factors, such as higher preoperative linguistic or cognitive abilities, may prevent the decline of FA of the AF,^35,77-79^ which likely subserves the improved speech perception scores with the CI, as highlighted by the present results. This model gains further support from evidence linking verbal fluency, syntactic processing and speech in noise with FA measures of the left AF,^72,80,81^ as well as from studies demonstrating the role of the FA in executive functions, such as memory and attention.^70,82,83^

Notably, the correlation between FA in the left AF and speech perception was stronger at 6 months than at 12 months. Consistently, inclusion of the FA value of cluster AF1 in the linear regression model showed a weaker contribution to WRS at 12 months and no significant contribution to AVs at 12 months. This may suggest that high preoperative FA facilitates faster early adaptation to the CI, while later gains could reflect a slower experience-driven WM reorganization. Indeed, previous work has shown that even short-term cognitive/motor interventions, such as meditating, juggling or singing, can induce structural WM changes.^72,84^ However, even though functional brain modifications have been demonstrated following CI rehabilitation,^85^ detecting structural brain modifications post-CI remains very challenging due to massive imaging artefacts on MRI, especially for DWI.

In our previous structural GM analysis of a similar patient group, CI outcomes were associated with GM volume and cortical thickness of key language-related regions, including the left inferior frontal gyrus, the superior and middle temporal lobes and the inferior parietal cortex.^35^ The current results show that CI outcomes are also correlated with microstructural features of the left AF, which connects the previously reported cortical regions. Similarly, Phillips *et al*.^81^ reported such associations between FA of the AF and cortical thickness in language-related areas, including the auditory cortex (BA41–42), Wernicke’s area (BA22), the inferior parietal region (BA39–40) and Broca’s area (BA44–45). In summary, reduced GM volume or thickness in language-related regions is associated with lower anisotropy of the connecting WM tracts, resulting in poorer CI outcomes.

### The right inferior fronto-occipital fasciculus supports audiovisual speech perception with the cochlear implant

AVs at 6 months correlated significantly with both localized FA and streamline counts within the rIFOF. The IFOF links occipital and temporo-basal regions to the frontal lobe through the external capsule.^67^ The IFOF is involved in non-verbal semantic processing, as highlighted by its involvement in visual association tasks,^86^ as well as in object recognition and face perception.^87^ While the left IFOF is predominantly involved in speech semantic processing within the ventral language stream,^65-67,70,80^ the rIFOF supports sensory multimodal integration, including audiovisual and visuo-spatial processing.^88,89^ This hemispheric specialization is facilitated by the higher density of projections to the superior parietal lobe for the rIFOF, while the left IFOF exhibits a denser innervation of Broca’s area within the inferior frontal gyrus, involved in language.^88^ Moreover, the involvement of the rIFOF in semantic processing appears to scale with cognitive demand, for instance in tasks requiring access to working memory.^90,91^ Accordingly, it is not unexpected that the FA of the rIFOF would prove to be associated with enhanced AVs outcomes following CI, as highlighted by our results. This relationship is especially relevant in the context of HL, where the degradation of auditory input necessitates increased cognitive engagement to support compensatory multisensory integration.

### The corpus callosum supports audiovisual speech perception with the cochlear implant

The whole-brain analysis revealed two clusters within the right CC, namely CC1 in the splenium (forceps major) and CC2—spanning the body of the CC and internal capsule close to the sensorimotor area—with positive correlations between FA and AV speech perception after CI. Within the splenium, the number of streamlines was also positively correlated with AVs at 12 months. Additionally, higher mean FA in the genu—the anterior CC segment connecting bilateral frontal regions—was linked to better AVs outcomes after 6 months of CI use.

There is ample evidence that the splenium and the body of the CC are particularly affected by HL.^20,22,25,26,29,30^ In one prior report of patients with congenital deafness, better CI outcomes were associated with higher FA of the genu.^34^ Unlike in certain previous studies,^20,92^ we found no association between these regional FA values and either age of HL onset or its duration.

It is not surprising that FA of the CC correlated more strongly with AV speech perception than with auditory-only speech perception. Indeed, since speech is mostly processed within the left hemisphere, literal comprehension is generally robust to CC damage.^93^ In contrast, the CC is necessary for understanding non-literal and emotional aspects of language, which are processed within the right hemisphere,^93^ as well as for audiovisual speech comprehension, which entails multisensory interactions between primary sensory cortices (visual and auditory), as well as association areas of both hemispheres.^94-99^ Furthermore, the CC plays an important role in cognition^89,100^ and in the execution of complex tasks.^97,101^ Bilateral integration becomes increasingly important in older adults, who come to rely more extensively on bilateral neural recruitment.^102^ Since FA changes in the CC can predict cognitive decline,^103^ finding decreased FA in the CC due to acquired HL may foreshadow accelerated cognitive decline and ensuing difficulties in accomplishing highly demanding cognitive tasks.^20^

The association between the splenium and audiovisual processing was expected, given its established role in the interhemispheric transfer between the posterior temporal and occipital regions involved in audiovisual integration. In contrast, the relationship between audiovisual language function and the more anterior callosal segments, the body and genu, may be more surprising. In our whole-brain analysis, the CC2 cluster was situated in an area spanning the body of the CC and internal capsule near the sensorimotor cortex. As the body links the sensorimotor cortices, it aligns with evidence that AV speech processing also engages the motor and somatosensory regions involved in speech production.^96^ For instance, mirror neurons in the motor cortex are activated both during speech production and while observing speech-related movements, such as lip-reading.^96,104^ Furthermore, the somatosensory cortex, including Brodmann areas 2 and 3, plays a role even in auditory-only speech perception.^105^ Indeed, both functionnal MRI (fMRI)and electrophysiological studies have demonstrated somatosensory cortex activation during articulatory tasks.^106-108^ Additionally, speech perception can be influenced by haptic information, such as tactile cues obtained by placing a hand on the speaker’s mouth or jaw.^109-111^

Our tractography findings go in the same direction. Preoperative mean FA of the genu correlated positively with AVs at 6 months. This observation extends previous evidence linking frontal cortical thickness to CI outcomes^35,112^ and suggests that interhemispheric frontal connectivity may play a complementary role in speech recovery with a CI. The genu supports the integration of higher-order cognitive functions, and its structural integrity has been linked to attentional control, processing speed and verbal fluency.^113-116^ These processes facilitate speech perception in challenging listening environments, particularly when auditory input is degraded as in CI users. Thus, preserved genu microstructure may reflect more efficient cognitive and linguistic network integration, which in turn supports more effective audiovisual processing with a CI.

### Negative correlation between fractional anisotropy of the cortico-spinal tract and auditory speech perception

An unexpected finding was the ‘negative’ correlation between the FA values of the CST and speech perception outcomes at 12 months. Nevertheless, one earlier study reported a negative correlation between verbal intelligence quotient and verbal association with FA of the right CST, the left inferior frontal gyrus and the left SLF.^117-119^ Such observations suggest that a decrease in FA value does not necessarily imply WM disorganization, but may rather indicate intricate microstructural changes in relation to myelination or intra- or extracellular water composition,^19^ not necessarily to the detriment of brain function.^119^

Inherent limitations of DWI technology, including its unfitness to resolve crossing fibres, could also contribute to such counterintuitive findings. Indeed, by modelling one single mean direction per voxel, DWI analysis can lead to misinterpretation in instances of crossing or fanning WM fibres.^19,120,121^ The observed negative correlation between FA and CI outcomes may reflect a greater presence of crossing fibres within these voxels.

### Effects of handedness, sex and age on fractional anisotropy

The linear regression analysis revealed distinct effects of handedness, age and sex on FA across clusters. Left-handed and ambidextrous individuals exhibited lower FA in the left CST compared to right-handers, in keeping with prior findings.^122,123^ Cluster CC2 demonstrated higher mean FA in females compared to males, which is consistent with the stronger interhemispheric connections in females.^124^ Furthermore, the present results demonstrate that age-related FA decline differs with sex within clusters AF1 and CC2, also in accordance with previous literature.^117,119,125-127^ The GM/WM alterations related to HL also differ according to sex.^128^ It is noteworthy that the functional implications of analogous FA changes may likewise differ according to sex. For instance, lesser FA within the SLF in healthy ageing has been associated with poorer verbal fluency in males, but better performance in females.^119^ Such findings emphasize the intricate relationship between brain structure, function and sex. Interestingly, hormones and sex chromosomes independently contribute to distinct structural and functional differences in the mouse brain.^129^

### Limitations and future research

Our diffusion imaging data were acquired using a single-shell diffusion model. Multi-shell diffusion imaging would have allowed the application of more advanced microstructural models and yielded a more refined analysis of preoperative WM architecture.

Furthermore, although we found significant correlations between FA in several WM tracts, including the AF and CC, and speech perception outcomes after CI, the mechanisms underlying these associations remain speculative. We proposed that such relationships may be mediated by individual cognitive or linguistic abilities, a hypothesis that would require confirmation through a study with prospective cognitive assessments.

We did not assess whether cross-modal plasticity affected CI outcome as was recently shown,^130^ and our assessment of cognitive functions prior to the implant was rather basic. Finally, we did not consider vestibular function in our analysis. Yet, compensatory mechanisms for vestibular dysfunction, which is commonly associated with deafness, can affect structure and WM organization.^131^

## Conclusion

This is the first study to demonstrate that speech perception following CI in adults with postlingual HL correlates with preoperative DWI metrics. The left AF emerged as a key structure, with higher FA values associated with better auditory and AVs after 6 months of CI use. Additionally, attainment of effective audiovisual speech processing was linked to higher FA within the CC and the rIFOF. Cognitive factors, particularly linguistic abilities, may contribute to the preservation of WM integrity within these tracts, potentially counteracting the negative effects of HL. Such preservation could, in turn, support rehabilitation outcomes after CI. This hypothesis warrants further investigation in future research and should be considered in clinical assessments. Importantly, the predictors of short- and mid-term CI outcomes partially differed, suggesting that distinct mechanisms may underlie early adaptation and longer-term rehabilitation following implantation. While factors such as lower depression scores and greater cortical thickness of the left pars opercularis may facilitate rapid adaptation during the first months of CI use, their influence may decrease over time as CI-driven neuroplastic reorganization progressively reduces initial interindividual differences.

Sex strongly influenced WM microstructure and modulated age-related changes, highlighting the need to account for this and other demographic variables in DWI analyses. The potential for hearing experience to reshape WM microstructure over time also warrants further investigation, especially in the context of post-implantation neuroplasticity. Future studies integrating DWI with more refined cognitive assessments will be crucial to further our understanding of the neural substrates underlying speech perception in CI users.

## Supplementary Material

fcag373_Supplementary_Data

## Data Availability

The data supporting the findings of this study are available from the corresponding author upon reasonable request. The imaging data are not publicly available because they contain information that could compromise participant privacy and confidentiality. Code used to analyse the data is available at https://github.com/Hyedryn/elikopy.
