# Supplementary material for: White matter microstructural biomarkers predict cochlear implant outcomes in adults with postlingual deafness
Source: Brain Commun. 2026 Oct 8;8(5):fcag373. doi: 10.1093/braincomms/fcag373 (PMC13711672; doi:10.1093/braincomms/fcag373)
Supplement: Supplementary file 1 [file fcag373_supplementary_data.pdf]

Supplementary table 1

Supplementary table 1: Results of the Linear Regression Models estimating AVs post-CI

	Model Summary				Coefficients				
	Adj. R^2	SEE	ANOVA F	ANOVA P		B	SE B	t	P
**AVs 6M**	0.564	0.1134	8.958	<0.001	(Constant)	0.116	0.230	0.505	0.617
					Age	0.002	0.001	1.177	0.247
					Depression	-0.004	0.006	-0.691	0.494
					HT non-CI side	-0.003	0.001	-2.619	0.013
					WRS w/ HA	0.050	0.230	0.354	0.726
					AVs	0.085	0.105	0.806	0.425
					CT left pars op	0.171	0.047	3.613	0.001
					FA cluster AF1	0.570	0.215	2.654	0.012
Y = 0.116 + (0.002 x age) + (-0.004 x depression) + (-0.003 x HT non-CI side) + (0.050 x WRS w/ HA) + (0.085 x AVs) + (0.171 x CT left op A44) + (0.570 x FA cluster AF1)									
**WRS 6M**	0.450	0.2082	6.729	<0.001	(Constant)	-0.438	0.368	-1.189	0.241
					Age	0.002	0.002	0.138	0.352
					Depression	-0.020	0.011	-1.818	0.076
					HT non-CI side	-0.002	0.002	-0.950	0.347
					WRS w/ HA	0.032	0.237	0.137	0.892
					AVs	0.419	0.182	2.300	0.026
					CT left pars op	0.068	0.076	0.897	0.375
					FA cluster AF1	1.346	0.374	3.596	0.375
Y = -0.438 + (0.002 x age) + (-0.020 x depression) + (-0.002 x HT non-CI side) + (0.032 x WRS w/ HA) + (0.419 x AVs) + (0.068 x CT left op A44) + (1.346 x FA cluster AF1)									

Abbreviations: AVs: audiovisual score, WRS: word recognition score, Adj. R^2: adjusted R^2; SEE: Standard Error of the Estimate; B: Unstandardized Coefficients; SE B: Standard Error of the Unstandardized Coefficients; DHA: duration of HA use, HT (non-)CI side: hearing thresholds at 0.5 kHz at (non-) CI side; CT left pars op: cortical thickness of the left pars opercularis, FA cluster AF1: mean FA of the cluster AF1 highlighted by the whole brain analysis

Supplementary figure 1

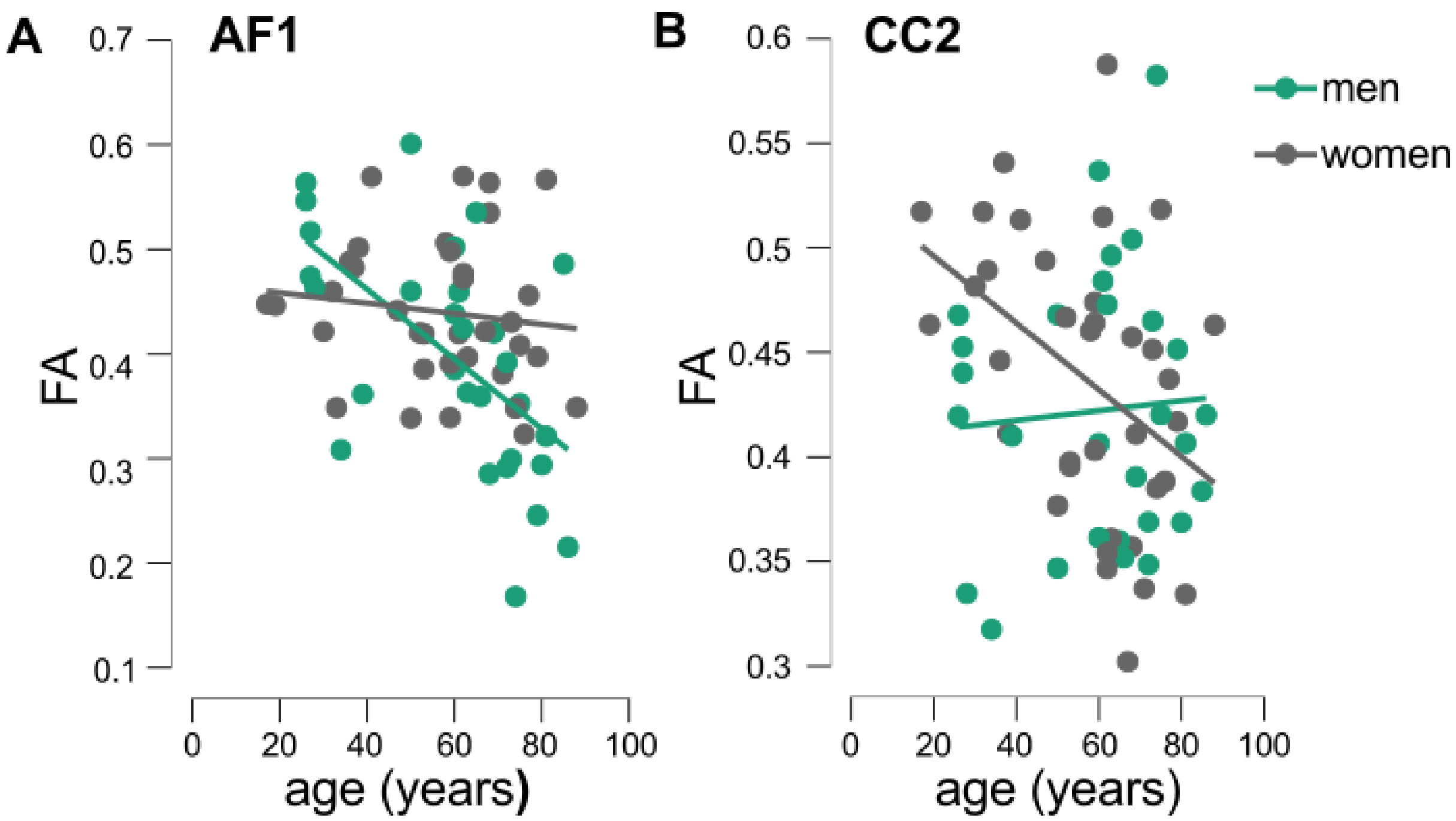

Sex-specific effects of age on FA in the arcuate fasciculus and corpus callosum. Scatterplots depict the relationship between age (x-axis) and fractional anisotropy (FA, y-axis) for males (green) and females (grey) in clusters AF1 (A) and CC2 (B). Each point represents an individual participant, and solid lines indicate the fitted linear regressions. In AF1, FA was significantly negatively associated with age in males but not females ($r = -0.585$, $p < 0.001$ vs. $r = -0.129$, $p = 0.453$). Conversely, in CC2, FA was significantly negatively associated with age in females but not males ($r = -0.422$, $p = 0.010$ vs. $r = 0.069$, $p = 0.722$). Analyses were based on $N = 65$ participants (29 males and 36 females).

Supplementary figure 2

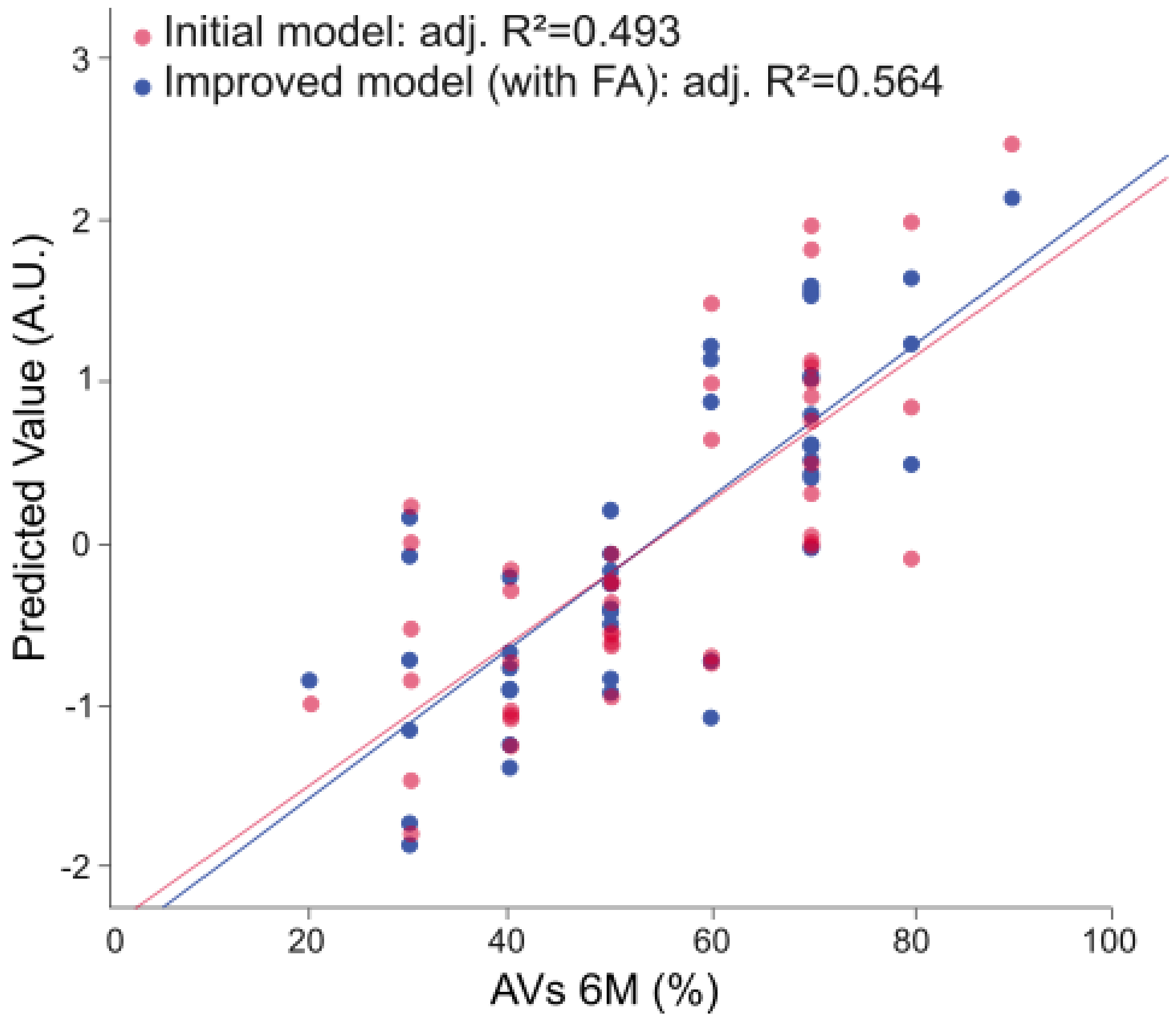

Inclusion of the FA of cluster AF1 improves estimation of CI outcomes. Linear regression models predicting audiovisual speech perception after 6 months of CI use (AVs 6M; x-axis) are shown against observed outcomes, with model-predicted values displayed on the y-axis. Each point corresponds to one participant, and lines represent the fitted regression relationships. The baseline model (red) was based on variables from a previously published prediction model. Incorporation of the mean FA of cluster AF1 (blue) significantly improved model performance ($p < 0.05$). Both models were fitted using data from N = 65 participants.
